# Astrocyte Activation in the Hippocampus Following Cardiopulmonary Bypass in a Rat Model: A Pilot Study

**DOI:** 10.7759/cureus.101465

**Published:** 2026-01-13

**Authors:** Kenji Yoshitani, Takahiro Tadokoro, Yusuke Nakano, Ryo Misawa, Hiroya Tsujimoto, Ayami Shinomiya

**Affiliations:** 1 Department of Transfusion, National Cerebral and Cardiovascular Center, Suita, JPN; 2 Department of Anesthesiology, National Cerebral and Cardiovascular Center, Suita, JPN; 3 Department of Anesthesiology, National Cerebral and Cardiovascular Center, Suitaa, JPN

**Keywords:** astrocyte, cardiopulmonary bypass, gfap, hippocampus, rat model

## Abstract

Background: Delirium after cardiac surgery is associated with poor outcomes. Cardiopulmonary bypass (CPB) increases blood-brain barrier (BBB) permeability and neuroinflammation, contributing to delirium. While microglial activation after CPB has been reported, the role of astrocytes remains unclear. This study aimed to determine whether astrocytes are activated in the hippocampus after CPB in a rat model.

Methods: Male Sprague-Dawley rats (13-15 weeks, 400-450 g) were anesthetized, mechanically ventilated, and randomized to sham (n=4) or CPB (n=4). CPB was performed with a primed circuit using a membrane oxygenator at 150 mL/kg/min for 60 min. Immunofluorescence and immunohistochemistry were performed to assess inflammatory markers and glial fibrillary acidic protein (GFAP) expression. GFAP-positive area density in the hippocampus was quantified, and groups were compared using the Mann-Whitney U test (p<0.05).

Results: GFAP immunofluorescence demonstrated increased astrocyte activation in the hippocampal CA3 region after CPB. Area density of GFAP-positive astrocytes was significantly higher in the CPB group than in the sham group (p=0.047).

Conclusion: CPB induced astrocyte activation in the hippocampal CA3 region. These findings indicate that CPB induces astrocyte activation in the hippocampus, consistent with a neuroinflammatory response.

## Introduction

Delirium following cardiac surgery significantly affects the survival rate of patients [1-4]. It has been observed that the use of cardiopulmonary bypass (CPB) enhances the permeability of the blood-brain barrier (BBB), which is associated with postoperative delirium [5]. Additionally, CPB may provoke neuroinflammation, which could be linked to the onset of delirium [6-9]. Disruption of the BBB allows circulating inflammatory mediators and immune cells to enter the brain parenchyma, thereby amplifying neuroinflammatory responses that are thought to contribute to postoperative delirium. Microglia play a crucial role in neuroinflammation, and reports indicate that microglia activate astrocytes, which are associated with increased permeability of the BBB [10]. Although microglial activation has been reported in a rat CPB model [11], the involvement of astrocytes in CPB-associated neuroinflammation has not yet been directly examined. The hippocampus is particularly vulnerable to systemic inflammation and BBB disruption, and hippocampal dysfunction has been implicated in postoperative cognitive disturbances. Within the hippocampus, the CA3 subregion plays a critical role in memory processing and is known to be sensitive to inflammatory and ischemic stress. Accordingly, focusing on astrocyte activation in the hippocampal CA3 region may help to characterize early, region- and cell-type-specific neuroinflammatory responses induced by CPB. Based on these observations, we hypothesized that not only microglial activation but also astrocyte activation occurs after CPB. Therefore, we conducted this preliminary study to investigate whether astrocytes are activated in the hippocampus following CPB in a rat model.

## Materials and methods

The experimental protocol was approved by the Animal Ethics Review Committee of the National Cerebral and Cardiovascular Center, Suita, Japan. All procedures met the guidelines for the proper conduct of animal experiments (Science Council of Japan, 2006).

Experimental procedures

Fasted male Sprague-Dawley rats (13-15 weeks old; weight, 400-450 g) were anesthetized with 5% isoflurane in oxygen in a plastic induction box. Once anesthesia was induced, the trachea was intubated with a venous 14-gauge catheter (Nippro, Osaka, Japan), and the lungs were mechanically ventilated using a small animal respirator (SN-480-7; Shinano Manufacturing, Tokyo, Japan). The ventilation parameters were set to a tidal volume of 10 mL/kg and a respiratory rate of 55-65 breaths/minute, ensuring that arterial PaCO₂ levels were maintained between 36 and 42 mmHg. Anesthesia was sustained with 1.5%-2.0% isoflurane, 2 µg/kg fentanyl, and 0.5 mg/kg rocuronium via the tail artery. Rectal temperature was monitored using BWT-100A (BRC Bioresearch Center, Nagoya, Japan) and servo-regulated to a target temperature of 37.0°C during CPB using a heating blanket and a convective forced air heating system. An Angiocath™ IV catheter (24G; Becton, Dickinson and Company; Franklin Lakes, NJ, USA) was placed in the right femoral artery to measure mean arterial blood pressure (BP). The ventral tail artery was cannulated with a 22-G, 28-mm IV catheter, which subsequently served as arterial inflow from the CPB circuit. A 4F multi-orifice venous cannula (modified from a 4-French Goodtec Sheath Introducer; NIPRO, Osaka, Japan) was advanced via the right external jugular vein for venous return. Heparin (150 IU) was administered after placement of the first arterial cannula, with an additional 150 IU administered just before placement of the jugular venous cannula. All cannulas were secured in situ with silk ties to eliminate the entrainment of extraneous air. The CPB group (n = 4) received a gas mixture of 40% oxygen (O₂) and air using a membrane oxygenator. Isoflurane (2.0%) was administered via gas inflow from the membrane oxygenator during CPB. The lungs of the animals were ventilated at a respiratory rate of 10-20 breaths per minute, ensuring that arterial PaCO₂ levels were maintained between 36 and 42 mmHg during CPB. The sham group underwent the same cannulation procedures but did not undergo CPB initiation. These animals were maintained in this state for 60 minutes, corresponding to the duration of the CPB procedure.

Cardiopulmonary bypass

To avoid excessive hemodilution, the bypass circuit was primed with approximately 22 mL of crystalloid, Voluben, and 6% hydroxyethyl starch (Otsuka Pharmaceutical Factory Inc., Tokushima, Japan). The CPB circuit consisted of a specifically designed 4 mL venous reservoir (modified circuit for dialysis, JMS, Osaka, Japan), a roller pump (MP600; NIPRO, Osaka, Japan), and a custom-designed small-volume oxygenator made of silicone fiber, providing a gas exchange surface area of 3.9×10⁻² m² (Fuji Systems, Tokyo, Japan). The circuit was warmed using a portable body warmer to avoid excessive heat loss. The entire circuit was primed with 22 mL Voluven. The rat CPB model employed in this study was established based on the model developed at Duke University [12]. All parts were connected using single-use silicone tubing. During CPB, the ventilation rate was lessened to 5-10 breaths/minute, and 1.5%-2.5% isoflurane was administered through the oxygenator. CPB was performed for 60 minutes at a flow rate of 150 mL/kg/min, adjusted to maximize the flow and maintain an optimal venous reservoir blood level. The wounds were sutured after the cannulas were removed.

Immunofluorescence

Immunofluorescence was performed to detect inflammatory markers in the hippocampus. Rats were deeply anesthetized with isoflurane and sacrificed immediately after completion of CPB. Animals were transcardially perfused with phosphate-buffered saline (PBS) followed by 4% paraformaldehyde (PFA). Brains were removed, post-fixed in the same fixative for 48 hours at 4°C, and then immersed in 30% sucrose solution for cryoprotection. Brain tissues were embedded, and coronal frozen sections (20 μm) were prepared.

Free-floating sections were washed with PBS and incubated overnight at 4°C in blocking solution containing Cy3-conjugated mouse monoclonal anti-glial fibrillary acidic protein (GFAP) (1:500; Sigma-Aldrich, catalog no. C9206). After washing with PBS, nuclei were counterstained with 4′,6-diamidino-2-phenylindole (DAPI) for 15 minutes. Sections were then rinsed, mounted with anti-fade mounting medium, and coverslipped. Sections containing the hippocampal CA3 region were selected for immunofluorescence analysis.

The slices were then washed with PBS, and an anti-fade mounting medium was added. Brain tissue slices were observed using a Keyence microscope BZX800 (Keyence Inc., Osaka, Japan), and images were acquired.

Data analysis

In this study, we evaluated the increase in activated astrocytes by assessing GFAP staining in rat tissues. The area of GFAP-positive cells was divided by the area of the hippocampus to calculate the area density. Quantitative image analysis was performed using ImageJ (National Institutes of Health, Bethesda, MD, USA). In a preliminary experiment, the variation in the area density was substantial. Therefore, among the four slices that included the hippocampus, the slice with the smallest area density was selected for comparison between the sham and CPB groups. Statistical analysis was performed using a general linear mixed-effects model, with rat treated as a random effect. The model included fixed effects for group, time, and their interaction. For the comparison of GFAP-positive astrocyte area density between the two groups, the Mann-Whitney U test was used. A p-value of less than 0.05 was considered statistically significant. All statistical analyses were conducted using R version 4.5.1 (R Foundation for Statistical Computing, Vienna, Austria).

## Results

The Sprague-Dawley rats were divided into sham (n = 4) and CPB (n = 4) groups. Table 1 presents the physiological data, including BP, heart rate (HR), and body temperature (BT), measured in both the sham and CPB groups at different time points: pre-CPB, 10 minutes (CPB10), 20 minutes (CPB20), 30 minutes (CPB30), 40 minutes (CPB40), 50 minutes (CPB50), and post CPB. There was a significant difference between the sham and CPB groups at 20 and 30 minutes after the start of CPB. BT significantly decreased 30 minutes after the start of CPB compared with pre-CPB.

**Table 1:** Physiological data of the sham and CPB group Data are expressed as mean ± SD. Statistical analysis was performed using a general linear mixed-effects model, with rat treated as a random effect and group, time, and their interaction included as fixed effects. In the sham group, CPB10, CPB20, CPB30, CPB40, and CPB50 represent time-matched measurement points (minutes) corresponding to the same elapsed times as in the CPB group, without initiation of cardiopulmonary bypass. Post CPB indicates measurements obtained immediately after termination of cardiopulmonary bypass. Abbreviations: MAP, mean arterial pressure; blood pressure; HR, heart rate; BT, body temperature; CPB, cardiopulmonary bypass. a, p<0.05 between the CPB and sham groups; *, p<0.05 compared with pre-CPB.

		Pre-CPB	CPB10	CPB20	CPB30	CPB40	CPB50	Post CPB
MAP (mmHg)	Sham (n=4))	76±13	84±12	85±8	79±22	80±19	81±16	79±18
	CPB (n=4)	94±14	92±11	91±11	83±27	88±22	89±27	86±31
HR (bpm)	Sham (n=4))	181±61	190±69	207±63	210±71	193±56	191±59	191±59
	CPB (n=4)	255±30	242±24	229±25a	225±37 a*	240±34	237±28	240±42
BT (°C)	Sham (n=4))	36.2±0.9	36.3±0.9	36.4±0.6	36.5±0.6	36.7±0.5	36.6±0.5	36.5±0.5
	CPB (n=4)	35.0±0.4	36.3±0.5	36.4±0.6	36.6±0.7 *	36.8±0.6 *	36.4±0.6	36.3±0.4

Table 2 presents the aortic blood gas data for the sham and CPB groups. In the acid-base equilibrium relationship, the pH and lactate values were significantly more acidic in the CPB group 50 min after the start of CPB. Base excess (BE) changed to the acidic side 10 and 50 min after the start of CPB, and bicarbonate (HCO^3-^) changed to the acidic side 10 min after the start of CPB compared to the baseline. The BE showed a significant acidic change after 10 min compared to the sham group. PaCO_2_, which affects cerebral blood flow, did not change significantly between or within the two groups in this study.

**Table 2:** Aortic blood gas date of the sham and the CPB group Data are expressed as mean ±SD. Statistical analysis was performed using a general linear mixed-effects model, with rat treated as a random effect and group, time, and their interaction included as fixed effects. In the sham group, CPB10 and CPB50 represent time-matched measurement points (minutes) corresponding to the same elapsed times as in the CPB group, without initiation of cardiopulmonary bypass. Post CPB indicates measurements obtained immediately after termination of cardiopulmonary bypass. Abbreviations: HCO^3-^:bicarbonate; BE, base excess; CPB, cardiopulmonary bypass; CPB10, 10 minutes after the start of CPB; CPB50, 50 minutes after the start of CPB; Glu, glucose; Lac, lactate. p<0.05 compared with pre-CPB; a, p<0.05 between the sham and CPB groups. *, p<0.05 compared with pre-CPB.

		Pre-CPB	CPB10	CPB50	Post CPB
pH	Sham (n=4)	7.43±0.07	7.46±0.07	7.36±0.11	7.35±0.10
	CPB (n=4)	7.48±0.02	7.40±0.07	7.37±0.03*	7.43±0.10
PCO_2 _(mmHg)	Sham (n=4)	42±10	37±5	35±7	40±6
	CPB (n=4)	34±2	36±10	42±5	36±10
Lac (mmol/L)	Sham (n=4)	1.70±0.86	1.47±0.10	5.73±6.29	5.08±5.73
	CPB (n=4)	1.30±0.34	2.75±1.52	3.64±1.89*	3.96±1.78*
HCO^3-^(mmol/L)	Sham (n=4)	27.1±2.8	26.2±0.8	20.3±9.1	22.7±7.7
	CPB (n=4)	25.8±1.1	21.8±3.2*	24.0±2.4	23.3±2.5
BE (mmol/L)	Sham (n=4))	2.4±1.3	2.5±2.3	2.2±1.4	2.2±0.1
	CPB (n=4)	2.6±1.3	-2.9±2.4 a*	-1.4±2.2*	-1.0±2.1*
PO_2_ (mmHg)	Sham (n=4))	174±39	147±7	107±11*	143±45
	CPB (n=4)	188±35	122±62*	89±30*	289±55 a*
SO_2_ (%)	Sham (n=4))	99.4±0.5	99.4±0.2	97.8±1.2	98.7±0.9
	CPB (n=4)	99.7±0.2	97.2±2.8	94.7±4.3*	99.9±0.1
Na^+^ (mmol/L)	Sham (n=4))	136±2	139±8	136±1	137±1
	CPB (n=4)	136±3	135±7	139±1	138±3
K^+ ^(mmol/L)	Sham (n=4))	4.4±0.3	4.3±0.4	4.2±0.6	4.5±0.1
	CPB (n=4)	4.7±0.5	3.7±0.4*	4.6±0.5	4.9±0.7
Cl ^-^(mmol/L)	Sham (n=4))	100±1	104±9	103±4	102±4
	CPB (n=4)	99±1	102±6	104±2*	104±3*
Ca^++ ^(mmol/L)	Sham (n=4))	1.32±0.12	1.28±0.06	1.23±0.10	1.28±0.01
	CPB (n=4)	1.21±0.07	1.23±0.04	1.30±0.07	1.30±0.12
Glu (mg/dL)	Sham (n=4))	325±27.5	308±55.9	444±46.7*	409±66.7*
	CPB (n=4)	326±66.3	292±72.6	348±105	359±112
Creatinine (mmol/L)	Sham (n=4))	0.49±0.08	0.50±0.05	0.63±0.20	0.67±0.05
	CPB (n=4)	0.51±0.07	0.56±0.30	0.66±0.06	0.63±0.11
Hb (g/dL)	Sham (n=4))	13.6±1.2	12.1±1.6	9.3±6.2	9.7±4.8
	CPB (n=4)	14.0±3.2	6.90±2.8a*	8.1±2.8*	8.5±3.3*

As for oxygenation parameters, PO_2_ was lower than the baseline 50 minutes after cannulation in the sham group and during CPB in the CPB group, and oxygen saturation (SO_2_) was also significantly lower than the baseline 50 minutes after the start of CPB. Potassium ions showed a significant decrease in dilatancy 10 minutes after initiation in the CPB group.

The following changes were observed in the electrolytes: chloride ions were significantly elevated in the CPB group at 50 minutes after starting and after weaning from the cardiopulmonary system, compared to the baseline. Blood glucose levels were significantly elevated in the sham group at 50 minutes after cannulation and extubation compared to the baseline.

Hemoglobin levels were significantly lower in the CPB group than in the baseline group after the start of CPB and significantly lower than those in the sham group 10 min after initiation of CPB.

**Figure 1:**
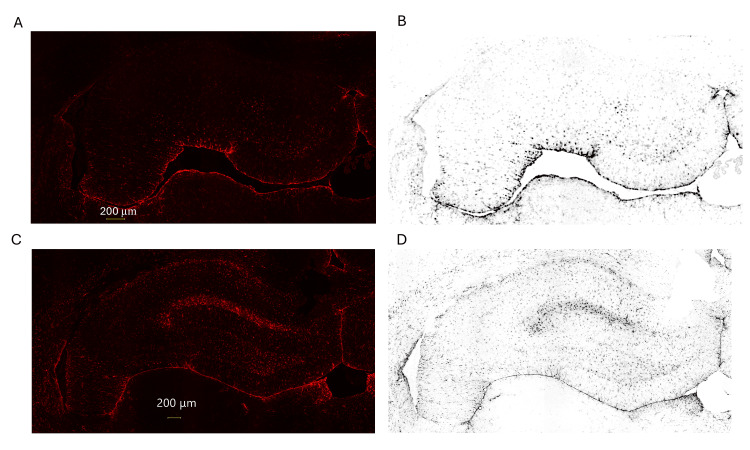
Representative GFAP immunofluorescence images and corresponding binary images for the quantification of astrocyte area density. (A, C) Immunofluorescence microscopy images of GFAP-positive astrocytes (red) in the hippocampal CA3 region from the same representative brain sections of the sham group (A) and the CPB group (C). (B, D) Corresponding binary images generated from the same regions of interest shown in panels A and C, respectively, and used for quantitative analysis of GFAP-positive area density. Scale bar: 200 μm (all panels). Note: For visualization purposes, the contrast of the binary images (B, D) was adjusted in this figure; all quantitative analyses were performed using the original, unmodified binary data. Abbreviations: GFAP, glial fibrillary acidic protein

The results of GFAP staining of positive cells, showing astrocyte activation, are shown in Figure 1. 

To assess astrocyte activation following CPB, we performed GFAP immunofluorescence staining in the hippocampal CA3 region of sham and CPB rats. Representative images of GFAP-positive astrocytes are shown in Figure 1. Fluorescence microscopy revealed a clear increase in the number and intensity of GFAP-positive astrocytes in the CPB group (Panel C) compared to the sham group (Panel A), indicating astrocyte activation following CPB.

To quantify astrocyte activation, we calculated the area density of GFAP-positive astrocytes by creating binary images from the original fluorescence images (Figure 1, Panels B and D). The area density was defined as the ratio of the GFAP-positive area to the total area of the hippocampal region. As shown in the binary images, the CPB group (Panel D) exhibited a significantly higher area density of GFAP-positive astrocytes than the sham group (Panel B) (p < 0.05).

**Figure 2:**
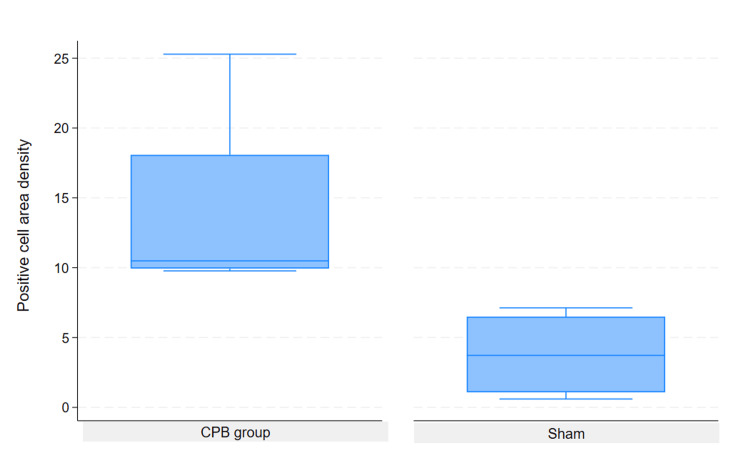
GFAP-stained astrocyte density in the hippocampus: a comparison between sham and CPB groups. Cardiopulmonary bypass (CPB) group, in which positive astrocyte density was measured following CPB; Sham group: Control group without CPB, in which astrocyte density was measured under baseline conditions. Statistical analysis was performed using the Mann–Whitney U test (*p < 0.05 vs. sham group).

Based on the Mann-Whitney U test results, there was a statistically significant difference between the two groups. The p-value for the two-tailed test (p = 0.0467) was less than the significance level of 0.05, indicating that the difference between the means of the two groups was significant (Figure 2).

## Discussion

In this study, we examined the effects of CPB on astrocyte activation in the rat hippocampus. Our results demonstrated an increase in GFAP-positive astrocytes in the CA3 region following CPB, suggesting that CPB triggers astrocyte activation as part of a neuroinflammatory response. These findings support the concept that astrocyte activation may contribute to BBB dysfunction and postoperative neurocognitive complications, such as delirium.

Astrocyte activation and neuroinflammation

The significant increase in GFAP-positive astrocytes in the hippocampal CA3 region suggests that CPB induces neuroinflammatory responses. Astrocytes are integral components of the neurovascular unit and play a critical role in maintaining BBB integrity [13]. Their activation can exacerbate BBB dysfunction, leading to increased vulnerability to neuroinflammatory processes [14].

Our findings align with emerging evidence linking BBB dysfunction to the pathogenesis of delirium. A recent comprehensive review by Gruenbaum et al. proposed that BBB disruption is a central mechanism underlying delirium, allowing neurotoxins and inflammatory mediators to enter the CNS [15]. Crucially, they identified GFAP not only as a marker of astrocyte injury but also as a key biomarker reflecting this BBB breakdown and subsequent neurocognitive decline. Our observation of increased hippocampal GFAP density provides histological support for this hypothesis in a CPB model, suggesting that the astrocyte activation we observed is a pathological step contributing to the BBB dysfunction associated with postoperative delirium.

Furthermore, this response is consistent with other CPB-related neuroinflammation studies. For instance, Wang et al. reported persistent microglial activation in a similar rat CPB model [11]. Given that activated microglia secrete cytokines that induce neurotoxic reactive astrocytes (A1 phenotype) [10], our results likely reflect the downstream effects of this glial interplay. Taken together, our data and these reports suggest that astrocyte activation is not merely a bystander effect but a potential driver of the BBB disruption and neuroinflammation that characterize CPB-associated cognitive dysfunction. However, given the pilot nature of the study, the small sample size, and the single time-point assessment, the present findings should be interpreted as descriptive and hypothesis-supporting rather than as definitive evidence of a causal mechanism linking CPB, astrocyte activation, BBB dysfunction, and postoperative delirium.

Limitations of the study

This study had several limitations. First, the sample size was relatively small (n = 4 for sham and n = 4 for CPB), which may have limited the generalizability of the findings. Therefore, the findings should be interpreted with caution, and further studies with larger cohorts are required to confirm these results. Second, astrocyte activation was assessed using a single immunohistochemical marker (GFAP) at a single post-CPB time point. As such, the present analysis provides a descriptive snapshot of astrocytic responses rather than a comprehensive or temporal characterization of neuroinflammation. Third, we did not perform behavioral or cognitive assessments; therefore, no direct association can be inferred between the observed histological changes and postoperative delirium or cognitive dysfunction. Additionally, while our study focused on astrocyte activation, we did not directly assess microglial activation or other inflammatory markers to further elucidate the neuroinflammatory pathways involved in CPB-related delirium. Future studies should incorporate a broader range of neuroinflammatory markers to provide a more comprehensive understanding of the cellular mechanisms involved.

Future directions

Further research is warranted to explore therapeutic strategies aimed at mitigating astrocyte activation and neuroinflammation during CPB. Anti-inflammatory therapies, neuroprotective agents, and strategies to maintain BBB integrity should be explored to prevent or reduce the incidence of postoperative delirium in patients undergoing cardiac surgery. Additionally, exploring the relationship between microglial and astrocyte activation could provide insights into the synergistic effects of these glial cells on neuroinflammatory processes.

## Conclusions

This study provides preliminary evidence that CPB is associated with increased GFAP immunoreactivity in the hippocampal CA3 region of rats, suggesting early astrocytic reactivity following CPB. Given the small sample size and the use of a single astrocytic marker, these findings should be interpreted with caution. Because GFAP upregulation typically reflects delayed astrocyte activation, future studies incorporating longer observation periods after recovery from anesthesia and CPB, additional markers of neuroinflammation and BBB integrity, and behavioral assessments will be essential to clarify the temporal dynamics and functional significance of astrocytic responses after CPB.
